# Lymphocytes in Placental Tissues: Immune Regulation and Translational Possibilities for Immunotherapy

**DOI:** 10.1155/2017/5738371

**Published:** 2017-11-19

**Authors:** Tom Erkers, Arwen Stikvoort, Michael Uhlin

**Affiliations:** ^1^Division of Blood and Marrow Transplantation, Department of Medicine, Stanford University School of Medicine, Stanford, CA, USA; ^2^Department of Oncology-Pathology, Karolinska University Hospital Huddinge, Stockholm, Sweden; ^3^Department of Clinical Immunology and Transfusion Medicine (KITM), Karolinska University Hospital Huddinge, Stockholm, Sweden; ^4^Department of Science, Intervention and Technology (CLINTEC), Karolinska Institutet, Stockholm, Sweden

## Abstract

Immune modulation at the fetomaternal interface is crucial to ensure that the fetal allograft is not rejected. In the present review, the focus is to describe basic functions of lymphocyte populations and how they may contribute to fetomaternal immune regulation, as well as determining what proportions and effector functions of these cells are reported to be present in placental tissues in humans. Also explored is the possibility that unique cell populations at the fetomaternal interface may be targets for adoptive cell therapy. Increasing the understanding of immune modulation during pregnancy can give valuable insight into other established fields such as allogeneic hematopoietic stem cell transplantation and solid organ transplantation. In these settings, lymphocytes are key components that contribute to inflammation and rejection of either patient or donor tissues following transplantation. In contrast, an allogeneic fetus eludes rejection by the maternal immune system.

## 1. Introduction

Immunological tolerance and defensive mechanisms of foreign tissue were first discussed by Murphy [1] and Owen [2]. However, the concept of acquired immune tolerance was introduced definitively by Billingham and Medawar in 1953 [3].

The sites at which the fetal and maternal tissues are in contact can be referred to as the fetomaternal interface and can be divided into two compartments. The first of which is between the maternal decidua and the fetal chorionic plate and chorionic membrane. Depending on whether the decidua is in contact with the site of implantation or with the fetal membranes is referred to as the decidua basalis or decidua parietalis, respectively. The second interface is where the maternal blood is in contact with the placental body and interacts with fetal trophoblasts. Thus, fetal and maternal tissues are not completely separated and immune cells have access to fetal tissues, driving complex tolerogenic immunological mechanisms to prevent rejection of the fetal allograft. The objective of this review is to discuss some of these mechanisms in the light of the current literature, with particular emphasis on lymphocyte function at the fetomaternal interface and how these cells may contribute to immune modulation during pregnancy.

## 2. T Cell Priming and Fetal Antigen Presentation

The placenta can be regarded as a haploidentical transplant. However, transplantation of a solid organ or hematopoietic stem cells leads to rejection or graft-versus-host disease (GVHD) without proper immunosuppressive interventions, while pregnancy is tolerated. Thus, there must be fundamental differences in these two entities in the priming and effector responses of the immune system to nonself. Acute graft rejection is driven by direct and indirect allorecognition [4]. Donor or recipient tissue-resident antigen presenting cells (APCs) collect graft antigens and migrates to adjacent lymphoid organs. Presentation of a foreign peptide to a T cell by a foreign APC elicits a stronger response in a larger quantity of T cell clones than if a foreign peptide is presented by self APCs [5]. Interestingly, studies have indicated that indirect allorecognition―and not direct allorecognition―is the major pathway for the maternal immune system to recognize fetal antigens [6, 7]. Using an Act-mOVA system [8], predominant maternal APC presentation of fetal antigens is suggested, as OVA-specific T cells respond to Act-OVA transgenic fetuses but not to fetuses deriving from control males [7, 9].

Moreover, trophoblasts have shown to have no expression of major histocompatibility complex (MHC) class II molecules, which limits the priming of CD4^+^ T cells by fetal cells in the placenta [10]. Trophoblasts also have expression of human leukocyte antigen- (HLA-) C, HLA-G, and HLA-E, while expression of the more polymorphic HLA-A and HLA-B is limited, resulting in a reduced recognition of alloantigens. HLA-C is the only classical HLA molecule expressed by fetal trophoblasts. Interestingly, a study with HLA-C mismatch between mother and father showed an increase in frequencies of CD4^+^CD25^dim^ T cells in decidual tissue [11]. Additionally, the placental tissues also contained CD4^+^CD25^high^ cells, supposedly regulatory T cells (Tregs). This was not seen in pregnant women when the mismatch was for HLA-DR or HLA-DQ.

Besides the restriction of indirect allorecognition for activation of T cells, studies have indicated that the dendritic cells resident in the decidua are constrained in their ability to leave the tissue and migrate to adjacent lymph nodes where they can activate circulating T cells [12]. Collins et al. have suggested that the dendritic cells (DCs) in the decidua are immobile despite being responsive to the chemokine CCL21, one of the ligands for CCR7 that enable homing to lymphatics. The DCs stay immobile even after being activated through exposure to lipopolysaccharides.

Lymphangiogenic molecules are produced by the first or second trimester *ex vivo* cultured invasive cytotrophoblasts. These cells have shown to stimulate lymphatic remodeling and growth of lymphatics when transplanted into an *in vivo* model [13]. Thus, the process by which cytotrophoblasts enable lymphatic remodeling may be important in implantation and vascularization [14]. In contrast, a later study showed that the lymphatics disappeared in human endometrium following decidualization [15], limiting the possibility of primed DCs to migrate to lymphoid organs. Interestingly, extravillous trophoblasts may enter decidual veins as early as week 5 of gestation [16, 17]. The implication of this for fetomaternal tolerance is not known. This does not rule out the possibility of fetal-derived peptides reaching the lymph nodes and being taken up by APCs. Exosomes containing fetal peptides are readily produced by placental tissues throughout pregnancy and may contribute to modeling immune responses and contribute to transport fetal peptides for antigen presentation [18]. To our knowledge, there are no studies investigating if these exosomes can contribute to partial central tolerance towards the fetus during pregnancy.

In addition to reduced DC migration for efficient T cell priming, reports are suggesting that gene silencing in the decidua prevents migration of effector T cells to the fetomaternal interface [19]. Specifically, upon activation by tumor necrosis factor- (TNF-) *α*, the myometrium upregulates the levels of transcription for RNAs encoding *Ccl5*, *Cxcl9*, and *Cxcl11* whereas stromal cells from the decidua do not. These genes encode chemokines that are ligands for CXCR3, which is present on Th1 cells and increase their homing towards tissues expressing these chemokines. Altogether, the results of these studies indicate that one way of maintaining fetomaternal tolerance is to limit allorecognition, inhibit APC migration to lymph nodes, and reduce infiltration of effector T cells.

## 3. Lymphocyte Cell Composition at the Fetomaternal Interface and Their Role in Tolerance

### 3.1. NK Cells

A major difference between a transplanted allograft and the fetomaternal interface is their different immune cell composition. The majority of immune cells present in these tissues are natural killer (NK) cells [20]. One of the known roles of these cells is to increase and modulate the vascularization between the placenta and the uterus to enable sufficient blood flow at the fetomaternal interface. Deficiencies in this process inhibits the growth of the fetus with adjacent tissues and may lead to preeclampsia [10, 21]. NK cell is also immunologically relevant in maintaining tolerance during pregnancy.

The NK cells found in the decidua during pregnancy are functionally and phenotypically different from conventional NK cells found in adults and children [20, 21] and are referred to as decidual NK (dNK) cells. Phenotypically, these cells have a very high expression of CD56. During the first trimester, dNK cells may comprise up to two thirds of the total lymphocyte repertoire [22]. NK cells in peripheral blood range between 5–30 percent and approximately 0–7 percent of the NK cells are activated [23]. Compared to conventional NK cells, dNK cells have an increased expression of natural killer group 2 (NKG2) receptors [24–26]. These receptors recognize antigens presented by HLA-C and HLA-E. Moreover, dNK cells have a high expression of killer cell lectin-like receptor subfamily D (KLRD1) [27], CD9 [28], and CD49a [29] and an increased frequency of expression of CD69 [22]. This shows that many dNK cells display a more activated phenotype. Indeed, the cytokine production in dNK cells is high [30] but despite having granules for cytotoxic ability, these granules fail to work efficiently and dNK cell cytotoxicity is therefore low [26]. There are a couple of different studies suggesting mechanisms for how the cytotoxic ability of dNK cells is impaired. Decidual macrophages can inhibit dNK cells cytolytic ability in a transforming growth factor- (TGF-) *β*-dependent manner [31]. The known roles of decidual macrophages in fetomaternal tolerance have been thoroughly covered in a review by Svensson-Arvelund and Ernerudh [32]. Furthermore, the cytotoxic ability of dNK cells is reduced by recognition of HLA-E expressed on trophoblasts [26, 33]. Additionally, dNK cells may interact with HLA-G^+^ extravillous trophoblasts (EVT). During the interaction of dNK cells and EVT, HLA-G can be acquired by the dNK cells, while during internalization and degradation, possible signaling of HLA-G leads to reduced cytotoxicity in dNK cells. However, dNK cell cytotoxicity can be increased in an inflammatory milieu [34, 35]. One study showed that those dNK cells in contact with cytomegalovirus-infected cells became cytotoxic following activation through NKG2C/D/E receptors [36]. Another study demonstrated dNK cells expressing the killer cell Ig-like receptor 2DS1 had cytotoxic function towards human cytomegalovirus presented by HLA-C2 on decidual stromal cells *in vitro* [37]. The secretory functions of dNK cells are distinctive from conventional NK cells *in vitro*. When stimulated with interleukin- (IL-) 15, dNK cells can produce interleukin- (IL-) 8, interferon-inducible protein- (IP-) 10, vascular endothelial growth factor (VEGF), placental growth factor [30], interferon- (IFN-) *γ*, and TNF-*α* [38]. These cytokines and growth factors are crucial for angiogenesis and arterial remodeling in early pregnancy.

IFN-*γ* is associated with Th1-like responses, including activation of macrophages, upregulation of MHC class I on APCs and epithelia. dNK cells may also contribute to modulating Tregs. IFN-*γ* induces production of indoleamine-2,3-dioxygenase (IDO) [39]. IFN-*γ* production by dNK cells can induce IDO expression in CD14^+^ cells in the decidua. When dNK cells were cocultured with CD14^+^ cells from the decidua and T cells, the frequency of Tregs was increased [40]. In the same setting, using conventional NK cells from the peripheral blood or only decidual CD14^+^ cells did not affect Treg frequencies. The frequency of Tregs in this system was also decreased when an anti-TGF-*β* antibody was added. Furthermore, decreased expression of killer immunoglobulin receptors (KIR) on NK cells in peripheral blood of women is associated with recurrent spontaneous abortions or unsuccessful implantation [41]. However, it is difficult to assess whether the shift in expression has implications in the implantation or in maintaining tolerance against paternal antigens. NK cell receptor activation leads to IFN-*γ* secretion through distinct pathways [42–44], and IFN-*γ* seems to be important for both implantation and to maintain tolerance during pregnancy. Investigating this axis would be interesting, especially with regard to expression of 2B4 and other coreceptors in dNK cells. This may give additional insight on the role of dNK cells in fetomaternal tolerance.

One study that used different toll-like receptor (TLR) stimulation on dNK could show differences in IFN-*γ* and IL-6 and TNF-*α* concentrations in supernatants when cultured *in vitro* [45]. The highest amount of these cytokines were produced when the cells were stimulated with TLR3 or TLR9. TNF-*α* production was favored when the cells were stimulated with TLR2, TLR3, and TLR9. dNK cells also showed to produce chemokines that may attract both granulocytes and T cells, including chemokine ligand (CCL) 5, IL-8, CCL3, and CCL4. NK cells in peripheral blood can produce trace amounts of IL-10 and TGF-*β* [46], but the translation of this regulatory NK cell subset in dNK cells or the presence of IL-10 producing NK cells in placental tissues has not been investigated to our knowledge.

A possible mechanism of immune regulation during pregnancy is through the downregulation of NKG2 on PBMCs [47]. This inhibits NKG2-dependent cytotoxic responses at the fetomaternal interface and is caused by production of MHC class I chain-related proteins A and B by syncytiotrophoblasts in the placenta.

### 3.2. B Cells

Certain maternal antibodies are known to be able to cross the placental barrier to provide a passive defense to the fetus [48, 49].

As early as the 1990s, a potential role for B cell frequencies in blood was identified. Maternal serum was shown to stimulate B cells to reduce production of IgM and increase production of IgA and IgE, while IgG production remained stable [50].

B cell activating factor (BAFF) is important for maturation of B cells [51]. A study by Lundell and colleagues can show that BAFF is produced by decidual stromal cells and BAFF levels are high at birth [52]. BAFF is induced in DSCs by interferon-*γ*/interferon*-α*, which can be produced by dNK cells. Increased BAFF levels in children are associated with a higher frequency of CD27^+^ memory B cells [53]. The same study also showed that infants with allergies at 18 months were associated with lower cord blood BAFF levels compared to infants with no allergic symptoms.

Changes in B cell subsets during pregnancy have also been linked to disease outcome in children postpartum. For instance, atopic asthmatic mothers were shown to have increased frequencies of transitional B cells (IgM^hi^CD38^hi^) as opposed to healthy pregnant mothers. Moreover, the atopic asthmatic mothers with the highest frequencies of transitional B cells were shown to have an increased risk of progeny with allergies [54]. Additionally, CD5^+^ B cells were shown to be reduced during pregnancy in the blood of healthy women, only to return to normal levels within 2 months' postdelivery. No difference was seen for other B cellular subsets [55].

B cells in the fetomaternal interface may protect against fetal rejection by asymmetric IgG production. This is thought to be influenced by Th2 cytokines produced from switching from a Th1 to Th2 phenotype in blood during pregnancy [56, 57].

Regulatory B (Bregs) cells are currently a focal point of research in the fetomaternal interface. In a recent review by Guzman-Genuino and Diener, the role of Bregs in transplantation, cancer, autoimmunity, and pregnancy are discussed in detail. IL-10 producing Bregs are of special interest in the pregnancy context, as IL-10 can be detected at heightened levels in the fetomaternal interface and is thought to counterbalance the proinflammatory response associated to fetal rejection [58]. The role for IL-10 in pregnancy can however be debated, as *in vivo* models have shown that successful pregnancy can be IL-10 independent [59].

### 3.3. *γδ* T Cells

Another cell population that has been inadequately discussed regarding a role during gestation and fetomaternal tolerance is *γδ* T cells. These cells constitute 5–10% of circulating T cells in adult peripheral blood and represent a bridge between innate and adaptive immunity [60]. The frequency of these cells is similar in decidua [61]. In umbilical cord blood, *γδ* T cells are less frequent than in peripheral blood, representing less than 1% of the lymphocytes [62]. Additionally, *γδ* T cells in umbilical cord blood are more naive and show a polyclonal repertoire with predominance of the V*δ*1 subtype in contrast to peripheral blood where V*δ*2 is in abundance [63]. *γδ* T cell function and their specific phenotype in the human placenta and at the fetomaternal interface have been described to some extent. Earlier studies suggest that *γδ* T cells in the decidua are skewing the immune response towards Th2 at the maternal-fetal interface [64, 65]. This is mediated by the production of IL-10 and TGF-beta [65, 66]. The importance of decidual *γδ* T cells for Th2 skewing via cytokines was later confirmed by Fan et al. [67] which also showed that the cells enhance trophoblast growth and invasion. This implies their dual role in both modulating the immune response in favor of fetomaternal tolerance as well as placental growth. What complicates the interpretation of the role of *γδ* T cells is that their phenotype differs greatly between the parietalis and basalis. In the former, more than 50% express V*δ*1, while the latter more resembles peripheral blood with more than 90% expressing V*δ*2 [68]. Studies on peripheral blood comparing healthy pregnant women and women at risk of premature pregnancy termination suggest that an increase in the V*δ*2/V*δ*1 ratio is associated with an increased risk of termination via a shift in Th1/Th2 balance [69].

### 3.4. Invariant Natural Killer (iNK) T Cells

iNKT cells are identified by expression of the complementarity-determining region 3 conserved invariant T cell receptor chain V*α*24-J*α*18 and the more diverse V*β*11 [70, 71] receptor chain. They are activated through interaction with glycolipids presented by the nonclassical MHC complex CD1d [72]. These cells are potent effector cells and can produce cytokines such as IFN-*γ*, IL-4, IL-13, TNF-*α* [73, 74], and IL-10 [75]. They also have cytotoxic ability [72], primarily through the Fas/FasL pathway [76].

The iNKT : T cell ratio is increased early following allogeneic hematopoietic stem cell transplantation in patients receiving conditioning with total lymphoid irradiation (TLI) combined with antithymocyte globulin (TLI) [77, 78]. These patients had a reduced incidence of GVHD. Moreover, a high number of iNKT cells in donor grafts are associated with of GVHD-free survival [79]. In a phase IIa trial, one third of the patients that received an iNKT ligand (RGI-2001) combined with sirolimus had increased frequencies of regulatory T cells [80]. However, it is difficult to assess the role of iNKT in this setting as SRL alone can promote Tregs [81, 82]. Murine studies further demonstrate the importance of iNKT cells for reduced incidence of GVHD and expansion of Tregs [83]. Thus, high numbers of iNKT cells in the allogeneic setting seem to be favorable for maintaining tolerance.

Comparatively, iNKT cells are highly enriched in the decidua compared to peripheral blood [84]. Upon stimulation with *α*GalCer or anti-CD3, decidual iNKT cells produced more IFN-*γ* and granulocyte macrophage-colony stimulating factor than IL-4 compared to iNKT cells from peripheral blood. Thus, it can be speculated that immunomodulation by iNKTs at the fetomaternal interface is directed towards functions associated with IFN-*γ*.

There are several murine studies linking iNKT function to pregnancy loss following administration of *α*GalCer [85–89]. *α*GalCer-stimulated pregnancy loss was associated with perforin when given early. In contrast, when given at later stages of pregnancy, it was associated with IL-2 and TNF-*α* [89]. This emphasizes the importance of different functions at different time points during gestation. Li et al. showed that administration of *α*GalCer reduces the number of decidual Tregs, as well as their ability to produce IL-10 and TGF-*β* [88]. The same study also shows that administration of both *α*GalCer and an anti-IFN-*γ* antibody restored Treg function *in vitro*. Although the majority of studies regarding iNKT cells during pregnancy indicates that iNKT cells may contribute to loss of tolerance and labor induction, there is a need for more studies exploring mechanisms for iNKT enrichment and function at the fetomaternal interface, especially in the human setting.

### 3.5. Innate Lymphoid Cells

Innate lymphoid cells (ILCs) can be divided into three groups: ILC1, ILC2, and ILC3. The ILC1 and ILC3 groups also have transcriptional, phenotypical, and functional heterogeneity [90–92]. Vacca et al. described the presence of ILCs in the decidua, showing that human first trimester decidua is host to two types of ILC3s [93]. One type was capable of producing IL-22 and IL-8, and the other one could secrete TNF-*α* and IL-17A. ILC1 cells were also present and produced IFN-*γ*. The same group also showed that ILC3s were present in the decidua in the proximity of neutrophils, suggesting that ILC3s contribute to the recruitment of neutrophils during the first trimester [94]. Moreover, coculture of ILC3s and decidual neutrophils increased survival of the neutrophils, whereas dNK cells could not. ILC3s were able to produce GM-CSF and CXCL8, which promote survival and migration in neutrophils. Thus, present data suggests that ILCs at the fetomaternal interface may contribute to microbial defense. Their role in fetomaternal tolerance is yet unexplored. A summary of findings in humans of lymphocytes discussed so far is presented in Table 1.

## 4. CD8^+^ T Cells

Compared to the NK and macrophage compartment, the number of T cells at the fetomaternal interface is low [95]. Compared to peripheral blood, where the T cell compartment contains more CD4^+^ cells than CD8^+^ cells, the fetomaternal interface contains more CD8^+^ T cells [61, 96, 97]. A recent study has shown that there is an accumulation of virus-specific effector memory (T_EM_) CD8^+^ T cells in the decidua during uncomplicated pregnancy, which may suggest that the skewing of the CD8^+^ T cell compartment may be to manage infections rather than allogeneic responses against fetal tissues [98]. CD8^+^ dT cells have been shown to be less cytotoxic than their counterpart in peripheral blood [99, 100]. These cells also appear to almost exclusively exert an effector memory phenotype [100]. Interestingly, despite having a lower expression of perforin and granzyme (GZM) B, the mRNA expression of these proteins are high [100]. Two studies from the Rao lab have presented data indicating that persistent IL-2 receptor *α* (IL-2R*α*) signaling can induce endoribonuclease dicer to produce microRNA to control perforin of CD8^+^ T cells during inflammation [101, 102]. The same studies also suggest that perforin transcription is regulated by signal transducer and activator of transcription 5 (STAT5) and eomesodermin [102]. We performed a study where we investigated how decidual stromal cells affected IL-2 production and IL-2R expression and signaling. We could observe that DSCs increased IL-2 production in an allogeneic setting *in vitro* [103]. This was followed by a high expression of the IL-2R*α* in CD8^+^ T cells. Furthermore, we showed that even though IL-2R*α* expression was high, the expression of the high-affinity IL-2R*αβγ*_c_ complex was reduced. This was associated with a significantly reduced IL-2 internalization and STAT5 signaling in CD8^+^ T cells. Thus, we suggest a hypothesis where DSCs induce a persistent high IL-2 production that through several pathways limits the effector function of CD8^+^ T cells. The increased production of IL-2 by DSCs may also contribute to the abundance of CD8^+^ dT cells with an effector memory phenotype, although the role of IL-2 in CD8^+^ T cell differentiation is difficult to determine [102, 104]. One recent study suggested that these cells have an increased expression of programmed death-1 (PD-1), indicating exhaustion as a possible mechanism with reduced effector function as a consequence [105]. We did not observe this when allostimulated CD8^+^ T cells were cultured with DSCs, but in the absence of DSCs and addition of equivalent concentrations of IL-2 as provided through DSCs, the cells expressed increased levels of PD-1 [103]. This indicates that DSCs contribute to reduce exhaustion in T cells, despite high concentrations of IL-2. How DSCs induces IL-2 is not known. A recent report by Fragiadakis et al. compared paired samples from maternal peripheral blood and cord blood at term and compared the evoked immune features upon stimulation with a cocktail of IL-2, IL-6, IFN-*α*2A, and granulocyte macrophage-colony stimulating factor (GM-CSF) [106]. Although the aim of the study is more directed towards peripheral immunity, they were able to show that T cells in maternal peripheral blood were hyporesponsive compared to T cells from cord blood. The study did not include immune cells isolated from the fetomaternal interface.

## 5. CD4^+^ T Cells

### 5.1. Th1 T Cells

A relatively high frequency of the T helper cell subset Th1 (identified by the transcription factor Tbet^+^ [107]) is present in maternal blood compared to umbilical cord blood at term [106]. These cells are characterized by their ability to produce IL-2, IFN-*γ*, and TNF-*β*, and can be differentiated following exposure of IL-12 [108]. Th1 cells (identified as CXCR3^+^CCR4^−^CCR6^−^ [109]) have been shown to be more abundant in the first trimester decidua compared to peripheral blood, while Th2 and Th17 ratios are lower compared to peripheral blood [110]. The study does not specify if the decidual lymphocyte cells were isolated from decidua basalis or decidua parietalis. This is in line with the high number of CD8^+^ T cells, where Th1 cells may be important for enhancement of the probability of activation of CD8^+^ cells [111], while Tregs support tolerance of the fetus. There has also shown to be a correlation between NK cells and Th1 priming [112]. The ability of dNK cells to prime Th1 cells at the fetomaternal interface is not known. Interestingly, this observation is in contrast to the study by Karjalainen et al., which showed in mice that decidual stromal cells (DSCs) may be able to use chemokine gene silencing to prevent CXCR3^+^ Th1 cells from reaching the placenta. There is also additional work in murine models suggesting that CXCR3 is involved in spontaneous preterm birth [113]. A study by Saito et al. showed that there was no significant increase of IFN-*γ* secreting cells in human peripheral blood during any trimester in pregnancy [114], whereas Matthiesen et al. showed increased numbers of IFN-*γ*^+^ and IL-4^+^ cells in all trimesters [115]. The disparity in results is probably due to differences in methods to detect these cells and that many cells can produce IFN-*γ*. However, the high ratio of Th1 cells in decidua is interesting as they secrete IFN-*γ*, although dNK is likely the largest producer of IFN-*γ* in this setting. IFN-*γ* activation leads to a broad transcription profile and diverse immunological functions [116, 117], including priming adaptive immune responses, inhibiting cell proliferation and inducing apoptosis [118]. IFN-*γ* is also important for production of IDO [119], which can be important for tolerance and induction of Tregs [120–123]. During uncomplicated pregnancy, concentrations of IFN-*γ* are low in the periphery and are significantly lower in the third trimester compared to healthy controls [124], but the concentration is nonsignificantly increased in patients with preeclampsia. The frequency of Th1 cells has shown to be increased in preeclampsia [114]. In women with multiple sclerosis (MS), symptoms are reduced during pregnancy and are associated with a decrease of Th1 cells [125, 126]. Interestingly, relapse of MS postpartum is associated with a continued decline of Th1 cells, while patients that remain in remission have restored Th1 values. Murine studies have shown that Th1 cells inhibit Treg induction during pregnancy and induce fetal loss [127]. The broad spectrum of function initiated by IFN-*γ* points towards an understanding that the regulation of IFN-*γ* is important during pregnancy and that the balance of IFN-*γ* may be important for maintaining fetomaternal tolerance. Inhibiting IFN-*γ* increases proliferation of allostimulated PBMCs that were otherwise suppressed by DSCs [120]. In contrast, DSCs suppress the secretion of IFN-*γ* in the same system [128]. Furthermore, pretreatment of DSCs with IFN-*γ* reduced their ability to inhibit alloantigen-induced proliferation [120].

### 5.2. Th2 T Cells

In contrast to Th1 cells, a Th2 phenotype is somewhat increased in peripheral blood during pregnancy and a Th2 predominant immunity has been thought to be associated with uncomplicated pregnancy [129]. Indeed, early studies have indicated that the cytokine profile at the fetomaternal interface was skewed towards Th2 [130]. However, as also stated by Saito et al. [129], the importance of Th2 cells in maintaining fetomaternal tolerance should probably be revised. There are several studies questioning the importance of Th2 cells for the maintenance of fetomaternal tolerance during pregnancy, and their role appears to be of less importance compared to other T helper effector cells. It is, however, important to also note that the proportions of these T cells can vary over time during pregnancy, and many studies focus in a set time point, mostly early pregnancy. Th2 cells are characterized by expression of the transcription factor GATA3 [131, 132] and are induced by IL-4 [133, 134]. The frequencies of Th2 (CCR4^+^CXCR3^−^CCR6^−^) cells have in one study been shown not to differ significantly in blood compared to the first trimester decidua [110]. Additionally, another murine study demonstrated that knockout of the Th2 effector cytokines IL-4, IL-5, IL-9, and IL-13 did not necessarily lead to fetal loss [135]. In a recent study investigating the transcriptional profile of human decidual lymphocytes, it was shown that the T cells in the decidua had a predominant Th1, Th17, and Treg profile [136]. In contrast, Kostlin et al. isolated granulocytic myeloid-derived suppressor cells (MDSCs) from human placenta. Where placenta-derived MDSCs were cocultured with PBMCs, they saw a shift towards a Th2 phenotype in an *in vitro* system [137]. Thus, the Th1/Th2 paradigm and its importance in fetomaternal tolerance still require exploration.

### 5.3. Th17 T Cells

Although a proportion of cells in the decidua have been shown to be associated with secretion of IL-17 [136], the frequency of Th17 cells is lower in the decidua compared to peripheral blood in the first trimester [110]. A similar study suggested that there is an increased frequency of IL-17 producing cells among lymphocytes in the decidua compared to peripheral blood [138]. The master regulator of Th17 cells is RORC [139], and the cells can be induced by IL-6, IL-1*β* [140], IL-21, and TGF-*β* [141–143]. Th17 cells have been associated with a wide range of inflammatory complications where mechanisms of central/peripheral tolerance are insufficient, including autoimmunity [144–146] and graft-versus-host disease [147, 148]. Elevated levels of IL-6 could be detected at the onset of spontaneous abortions [149]. Indeed, patients with recurrent miscarriage also had increased frequencies of CD4^+^ T cells expressing CCR6 and IL-17 [150]. A similar result was shown by Santner-Nanan et al., where the ratio of Tregs/Th17 was increased in healthy pregnancies compared to pregnancies ending in preeclampsia [151]. IL-17 produced by T cells has also been linked to contribute to inflammation in human amniotic mesenchymal cells by enhancing the production of IL-8 synergistically with TNF-*α* [152].

It is difficult to assess whether it is the Th17 cells that promotes inflammation in these settings, or if the results are a consequence of a reduction in Tregs. Tregs, or more specifically the transcription factor forkhead box P3 (FOXP3), suppress differentiation of Th17 cells [153] which in part can result in a Treg-dependent balance between Tregs and Th17 cells. Interestingly, IFN-*γ* has also been shown to suppress differentiation of Th17 cells from naive T cells [154], adding more complexity to the regulation of Th17 cells. In contrast to Th1 and Tregs, the mechanisms of Th17 function and immunity at the fetomaternal interface are yet relatively unexplored, including the role of Th17 cells for production of antimicrobial peptides [155] and recruitment of neutrophils [156].

### 5.4. Regulatory T Cells

The concept of cellular immunity was formed in the seventies and eighties when patients that received blood transfusions showed weaker responses to mitogenic stimuli *in vitro* [157, 158]. However, it would be more than a decade before Tregs were identified [159–162]. Treg function is dependent on expression of FOXP3 [160, 162]. Tregs have a high expression of CD25, cytotoxic T lymphocyte antigen- (CTLA-) 4 [163], and a low expression of IL7-R*α* [164–166]. Tregs can be divided based on origin and effector function [167]. They can either be generated in the thymus or be induced in the periphery. Tregs can be divided into central Tregs and effector Tregs. Effector Tregs have low expression CCR7 and CD62L, while nonactivated Tregs have higher expression of CCR7 and CD62L [167, 168]. Upon TCR, CD28, and IL-2 stimulation, central Tregs will develop an effector phenotype and can have suppressive functions. Tregs are critical for maintaining peripheral tolerance and reduced levels are associated with poor outcomes in pregnancy [169, 170]. Tregs have a central role for fetomaternal tolerance and are detected in increased frequencies at the fetomaternal interface. In comparison, the frequencies of Tregs in peripheral blood between pregnant and nonpregnant women are similar [110, 123, 171, 172]. Effector CD45RA^−^ Tregs from term decidua parietalis are increased compared to peripheral blood and the endometrium [173, 174]. This is not seen in the peripheral blood of second trimester women [175, 176].

Increased levels of Tregs have been shown in patients with miscarriages [177], and Mjosberg et al. observed that Treg frequencies were reduced in the second trimester as a possible result of hormonal changes [178]. Peripheral Tregs in the second trimester of women with preeclampsia had an increased expression of CTLA-4 and CCR4 [175].

Continuous TCR, CD28, and IL-2 stimulation is important for Treg expansion and survival [179–181]. *FOXP3* represses production of IL-2 by Tregs themselves [182] and thus Tregs are dependent on other cells to provide IL-2. In contrast to conventional T cells, which seem to accumulate a reduced sensitivity to IL-2 signaling following alloactivation, Tregs maintain a high expression of pSTAT5 following exposure to IL-2 [103]. A recent study by Chinen and colleagues showed that IL-2 induced STAT5 signaling facilitated suppressive Treg function independently of TCR activation [183]. An earlier observation by Szymczak-Workman et al. also showed that TCR signaling is not necessary for suppressive Treg function [184]. Interestingly, we demonstrated that DSCs altered T cell responsiveness by inducing a high production of IL-2. One may speculate that this mechanism may favor Tregs at the fetomaternal interface, as they are less sensitive to IL-2R depletion and can have suppressive function in presence of IL-2.

Tregs can produce IL-10 to suppress immune responses [185, 186] and are important in maintaining tolerance [187]. CTLA-4 is crucial for Treg function [188–190]. CTLA-4 binds to CD80/CD86 on APCs and through this, interaction induces indoleamine-2,3-dioxygenase (IDO) in the APCs [191, 192]. CTLA-4 has been shown to reduce the expression of costimulatory molecules on APCs [188, 193] by transendocytosis [194, 195]. The intrinsic effects of CTLA-4 on Tregs are not known and under investigation [196, 197]. Tregs may also use lymphocyte activation protein- (LAG-) 3 [198], CD39/CD73 [199, 200], inducing IL-10 and TGF-*β* production in APCs [201] and production of TGF-*β* [202] or IL-35 [203] by the Tregs for suppression.

While conventional T cells that respond to self-antigens are terminated during thymic selection, part of the naturally occurring Treg repertoire responds to self-antigens [204]. These Tregs are dependent on continuous TCR activation to maintain functionality [205]. Mapping the TCR repertoire at the fetomaternal interface and linking its reactivity to maternal and/or paternal antigens using similar assays [206, 207] could elucidate Treg and conventional/invariant T cell evolution and specificity at the fetomaternal interface. Kahn et al. showed in murine models that Tregs recognize paternal antigens and that the suppressive function of these Tregs is antigen specific [208]. In the same study, ablation of Tregs in pregnant mice using a fusion protein of IL-2 and diphtheria toxin [209] led to significantly reduced number of births and reduced fetal birth weight. Moreover, adoptive transfer of Tregs to a T cell-depleted murine model may reverse an otherwise high rate of fetal loss [210]. In a review by Erlebacher, the data regarding the antigen specificity of the Tregs at the fetomaternal interface is debated, as so far they only are able to show that fetal absorption rates increase in T cell-/Treg-depleted allogeneic *in vivo* models, whereas this is not the case in syngeneic models [6]. Thus, identifying patterns of repertoire and antigen disparity in successful and complicated pregnancies could lead to further progress in the understanding of maintaining a tolerogenic environment during pregnancy.

Induction of Tregs at the fetomaternal interface has also been associated to activity through the IDO and PD-L1 pathways [6, 211]. *In vitro*, inhibition of IDO activity reduces the frequency of Tregs in cultures with DSCs. This could not be seen when PD-L1 was neutralized [120]. One experimental study has shown that IL-10 is not necessarily needed for successful pregnancy [59] and neutralization of IL-10 does not influence stromal-induced inhibition *in vitro* [120], despite stromal cells from the decidua and umbilical cord are able to promote production of IL-10 [128]. In the same setting, neutralizing PD-L1, but not PD-L2, inhibits alloinduced proliferation [120]. These experiments suggest that many immunosuppressive functions overlap in order to maintain fetomaternal tolerance. The role of IL-10 in this setting seems to be of lesser importance if ablated alone. The immunomodulatory properties of cells originating from placental tissues still need further investigation. This is also discussed by PrabhuDas and colleagues [212]. Direct effector functions of Tregs in pregnancy and their importance for tolerance require further investigation. A summary of findings in humans regarding conventional T cells in pregnancy is shown in Table 2.

## 6. Lymphocytes from Placental Tissues for Adoptive Cell Therapy

**Figure 1:**
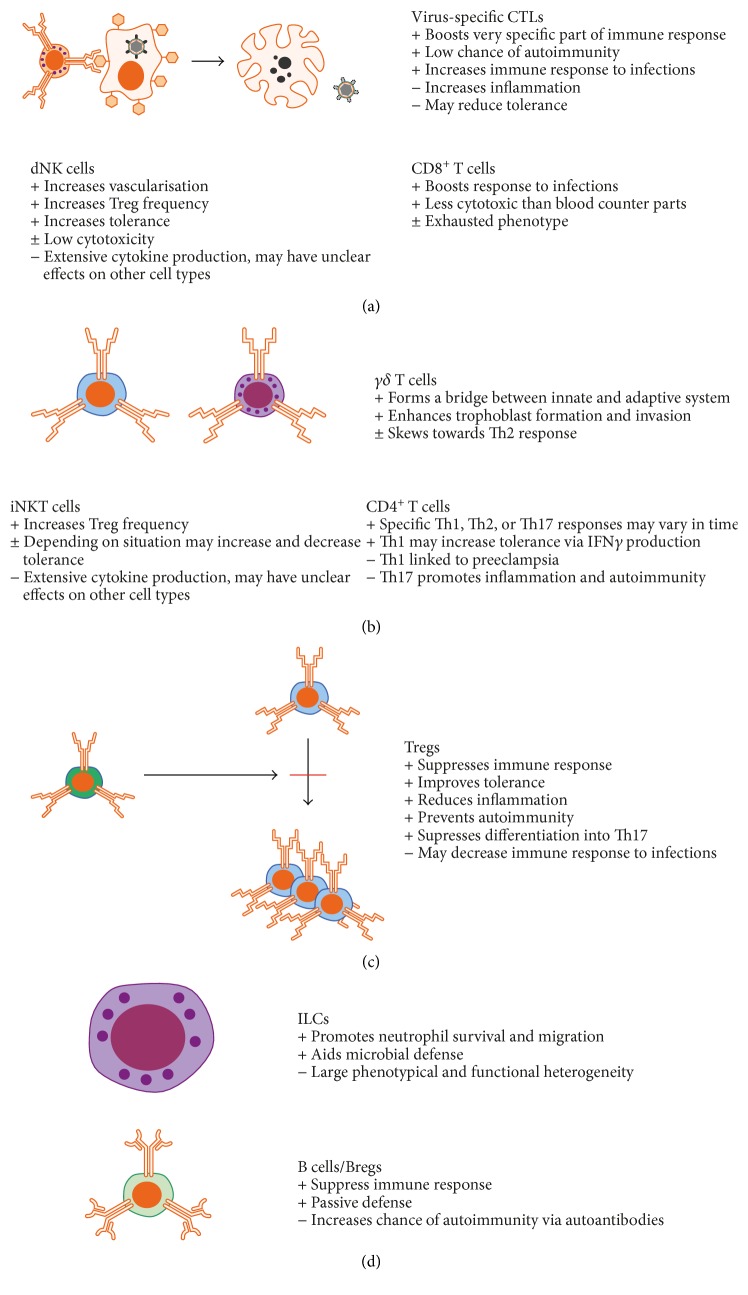
Summary of placental lymphocyte populations and features that may be of interest in the context of adoptive cell therapy.

The present review has discussed the heterogeneity of lymphocytes in placental tissues and their potential role in maintaining homeostasis during pregnancy. The main role of these cells in this context is summarized in Figure 1. As there has to be a completely different immune paradigm during pregnancy to maintain a low alloreactivity towards the fetus while ensuring sufficient protection towards infections, mechanisms for fetomaternal tolerance could be investigated for translational purposes in several areas. This includes improving tolerance and reducing inflammation following transplantation, prolonging remission or prevention of autoimmunity, or enhancing immunological responses towards infections. In all of these areas, lymphocytes are key components for determining outcome in patients. As the placenta is normally discarded following delivery, these tissues may provide a source for large amounts of primary effector cells that may be investigated in *ex vivo* studies.

The complexity of the to date identified mechanisms for fetomaternal tolerance highlights the difficulties of identifying effective diagnostic and treatment protocols to promote successful uncomplicated pregnancies. As we have discussed how lymphocytes exert effector functions at the fetomaternal interface, we can also identify unique cell populations that may be of particular interest for immunotherapy applications.

The extent of placental lymphocytes for cell therapy is limited to possible autologous applications. The reduction of MS symptoms during the third trimester identifies a potential patient group that may benefit by further investigations with the approach to decrease inflammation-induced neurodegeneration using cell therapy. One cell type that critically contributes to peripheral tolerance during pregnancy is Tregs. Tregs at the fetomaternal interface have shown in murine models to have response specifically towards paternal antigens [208]. An intriguing future study would be to investigate the extent of exosome-derived peripheral fetal antigen presentation during pregnancy [213]. This can later be used to determine if the reduction of MS symptoms in the third trimester is due to altered immune homeostasis in general or perhaps a local antigen-specific response by Tregs.

Moreover, the lymphocyte population in placental tissue that differs compared to their peripheral counterpart are mainly dNK cells. However, unpredictable plasticity of placental tissue-resident lymphocytes outside the unique placental compartment is not thoroughly explored and NK cell has a high plasticity [214]. NK cells expressing a single type of killer cell immunoglobulin-like receptor are under investigation as cell therapy towards cancer.

iNKT cells are also enriched in placental tissues. Just like NK cells, iNKT cells can be activated by different receptors present on the cells. Thus, specificity of these cells can be difficult to control. iNKT cells in humans also have diverse effector functions within subpopulations, indicating a high plasticity or a higher heterogeneity than presently described [73]. Nevertheless, iNKT cells are potent cytokine-secreting effector cells and the literature is associating small quantities of these cells with large biological impact [79].

Allogeneic donor T cell therapy is utilized in transplantation to prevent leukemic relapse. Okas et al. show that T cells from umbilical cord grafts can be expanded and used as donor lymphocyte infusions (DLI) [215]. A clinical study by the same group also showed the results of treatment with these cells. The small patient material limits evaluation of clinical benefit and safety. One of the patients developed GVHD, but it is difficult to assess if this was a direct consequence of the DLI [216]. The highly immunosuppressive state in transplantation patients is a prerequisite for the use of allogeneic T cell therapy. There are clinical trials showing that third party Epstein-Barr virus-specific T cells can be used as adoptive cell therapy [217]. T cells can be expanded a thousandfold and still maintain effector functions *in vitro* [218]. However, the benefit of isolating placental T cells compared from peripheral blood can be discussed, as autologous T cells of a high clonal diversity are present in peripheral blood and specific clones can be expanded *in vitro*.

## 7. Conclusion

The placenta is an interesting site of immune modulation which ensures survival of the fetus. We have discussed some of the mechanisms which may be of importance for immune tolerance during pregnancy.

As the placenta is normally discarded following delivery, it can provide an accessible source of large numbers of unique tissue-resident effector cells that can be investigated for cellular therapies; both for immune modulation and improving microbial defenses. Lymphocytes from cord blood are established for several cell therapy applications, primarily within HSCT. Although lymphocyte therapies are utilized successfully, future applications of placenta-derived lymphocytes other than from cord blood appear to be restricted to small patient groups and conditions. Cells with unique functions at the fetomaternal interface need more investigation for these purposes and possible applications still remain to be identified.

## Figures and Tables

**Table 1:** Proportions of lymphocytes in human placental tissue and findings ex vivo relating to outcome or lymphocyte function.

Cell	Compartment	Approximate abundance	Comment	Reference
NK cells	T1 Dec	50–70% of Dec cells	CD3-CD56^+(+)^	[22, 219, 220]
	T1 Dec basalis		Low cytotoxic function	[26]
	T1 Dec		↑ Tregs by IFN-*γ* → IDO	[40]
	T1 Dec basalis		TLR chemokine patterns	[45]

B cells	Term CB		↓ BAFF → allergy and decreased B cell maturation	[53]

*γδ* T cells	T1 Dec	5% of CD3^+^	Naive. V*δ*2 skewing	[61]
	Term UCB	1% of lymph		[62]

iNKT cells	T1 Dec	0.5% of CD3^+^ versus 0.01% in PB	Th1 cytokine bias	[84]

ILCs	T1 Dec		IL-17A and IL-22 ICS	[93]

BAFF: B cell activating factor; CD: cluster of differentiation; Dec: decidua; CB: cord blood; ICS: intracellular staining; IDO: indoleamine-2,3-dieoxygenase; ILC: innate lymphoid cells; lymph: lymphocytes; NK: natural killer; PB: peripheral blood; T1–3: trimester 1–3; UCB: umbilical cord blood.

**Table 2:** Proportions of T cells in human placental tissues or peripheral blood and findings ex vivo relating to T cell function or outcome during pregnancy.

Cell	Compartment	Approximate abundance	Comment	Reference
CD8^+^	T1 Dec	70% of CD3^+^	Reversed CD4/8 versus PB	[61, 96]
	Term Dec basalis/parietalis		Virus-specific viral control	[98]
	Term Dec basalis/parietalis		↓ perforin versus PB	[100]
	Term PB		Hyporesponsiveness	[106]
	Term DSC		Hyporesponsiveness	[103]

CD4^+^	T1 Dec	20% of CD3^+^		[61]
*Th1*	T1 Dec	15% of CD4^+^	CCR4^−^CXCR3^+^CCR6^−^	[110]
	T1, T2, T3, PP PB	112, 110, 156, 53/100000 lymph	IFN-*γ* secreting cells	[115]
	T1 → T3 PB	20 → 17% of CD4^+^	IFN-*γ*^+^ ICS	[114]
	T1 → T3 PB	1% lower CD4^+^CD45RA^+^	IFN-*γ*^+^ ICS, Rel free versus Rel MS PP	[125]
*Th2*	T1 Dec	5% of CD4^+^	CCR4^+^CXCR3^−^CCR6^−^	[110]
	T1, T2, T3, PP PB	84, 87, 119, 54/100000 lymph	IL-4 secreting cells	[115]
	T1→ T3 PB	2.3 → 3% of CD4^+^	IL-4 ICS	[114]
	T3 PB	3% versus 1.5% of CD4^+^	No PE versus PE	[114]
*Th17*	T1 Dec	1-2% of CD4^+^	CCR4^+^CXCR3^+^CCR6^+^, IL17^+^ ICS	[110] [138]
	T1 → T3 PB	1-2% of CD4^+^	IL-17^+^ ICS	[138]
	Preterm amniotic fluid, decidua		Il-17^+^ ICS Th17 Inflammation in hAMSC	[152]
	T1 PB		↑ IL-17/CCR6^+^ in spontaneous abortion	[150]
*Treg*	T1 Dec	2% of CD4^+^	CD25^high^FOXP3^high^	[110]
	Term Dec, PB		Dec ↑ CTLA-4^+^, HLA-DR^+^, CD69^+^ versus PB	[123]
	T1, T2, T3 PB	6.7, 10.9, 8.9% of CD4^+^	CD4^+^CD25^+^	[171]
	T2 PB	6 versus 7.5% of CD4^+^	FOXP3^+^ pregnant versus nonpregnant. Reduced by hormones	[178]
	Term DSC		↑ Tregs, partly by IDO	[120]

CD: cluster of differentiation; Dec: decidua; DSC: decidual stromal cells; hAMSC: human amniotic mesenchymal stromal cells; ICS: intracellular staining; IDO: indoleamine-2,3-dieoxygenase; lymph: lymphocytes; PB: peripheral blood; PE: preeclampsia; PP: postpartum; MS: multiple sclerosis; Rel: relapse; Treg: regulatory T cells; T1–3: trimester 1–3; TLR: toll-like receptor; UC: umbilical cord.
